# *DGKI* Methylation Status Modulates the Prognostic Value of *MGMT* in Glioblastoma Patients Treated with Combined Radio-Chemotherapy with Temozolomide

**DOI:** 10.1371/journal.pone.0104455

**Published:** 2014-09-18

**Authors:** Amandine Etcheverry, Marc Aubry, Ahmed Idbaih, Elodie Vauleon, Yannick Marie, Philippe Menei, Rachel Boniface, Dominique Figarella-Branger, Lucie Karayan-Tapon, Veronique Quillien, Marc Sanson, Marie de Tayrac, Jean-Yves Delattre, Jean Mosser

**Affiliations:** 1 CNRS, UMR 6290, Institut Génétique et Développement de Rennes, Rennes, France; 2 Université Rennes 1, Université Européenne de Bretagne, Biosit, Faculté de Médecine, Rennes, France; 3 Plate-forme Génomique Santé Biogenouest, Biosit, Rennes, France; 4 Centre Hospitalier Universitaire de Rennes, Service de Génétique Moléculaire et Génomique, Rennes, France; 5 Assistance Publique-Hôpitaux de Paris, Service de Neurologie 2 Mazarin, Groupe Hospitalier Pitié-Salpêtrière, Paris, France; 6 Département de Biologie Médicale, Centre Eugène Marquis, Rennes, France; 7 Centre Hospitalier Universitaire d'Angers, Service de Neurochirurgie, Angers, France; 8 Assistance Publique-Hôpitaux de Marseille, Centre Hospitalier Universitaire de la Timone, Service d'Anatomie Pathologie et de Neuropathologie, Université Aix-Marseille, Marseille, France; 9 INSERM U911, Université Aix-Marseille, Marseille, France; 10 INSERM U935, Poitiers, France; 11 Université de Poitiers, Poitiers, France; 12 Centre Hospitalier Universitaire de Poitiers, Laboratoire de Cancérologie Biologique, Poitiers, France; 13 Centre de Recherche de l'Institut du Cerveau et de la Moelle Épinière, INSERM UMRS 975/CNRS UMR 7225/Université Pierre-et-Marie-Curie, Institut du Cerveau et de la Moelle Épinière, Groupe Hospitalier Pitié-Salpêtrière, Paris, France; University Hospital of Navarra, Spain

## Abstract

**Background:**

Consistently reported prognostic factors for glioblastoma (GBM) are age, extent of surgery, performance status, *IDH1* mutational status, and *MGMT* promoter methylation status. We aimed to integrate biological and clinical prognostic factors into a nomogram intended to predict the survival time of an individual GBM patient treated with a standard regimen. In a previous study we showed that the methylation status of the *DGKI* promoter identified patients with *MGMT*-methylated tumors that responded poorly to the standard regimen. We further evaluated the potential prognostic value of *DGKI* methylation status.

**Methods:**

399 patients with newly diagnosed GBM and treated with a standard regimen were retrospectively included in this study. Survival modelling was performed on two patient populations: intention-to-treat population of all included patients (population 1) and *MGMT*-methylated patients (population 2). Cox proportional hazard models were fitted to identify the main prognostic factors. A nomogram was developed for population 1. The prognostic value of *DGKI* promoter methylation status was evaluated on population 1 and population 2.

**Results:**

The nomogram-based stratification of the cohort identified two risk groups (high/low) with significantly different median survival. We validated the prognostic value of *DGKI* methylation status for *MGMT*-methylated patients. We also demonstrated that the *DGKI* methylation status identified 22% of poorly responding patients in the low-risk group defined by the nomogram.

**Conclusions:**

Our results improve the conventional *MGMT* stratification of GBM patients receiving standard treatment. These results could help the interpretation of published or ongoing clinical trial outcomes and refine patient recruitment in the future.

## Introduction

Glioblastoma (GBM) is the most common and aggressive primary brain tumor in adults. Its prognosis remains extremely poor, despite multimodal treatment by surgery, radiotherapy, and temozolomide-based chemotherapy (standard regimen) [1]. The most consistently reported clinical prognostic factors for GBM are age, extent of surgery, and performance status [2], [3], [4]. The somatic mutation affecting amino acid 132 in the isocitrate dehydrogenases 1 gene (*IDH1*) is also associated with a better clinical outcome in gliomas, including glioblastoma. However, this mutation is rare in primary GBMs (approximately 6%) [5], [6], [7]. The methylation status of the O^6^-methylguanine DNA methyltransferase gene (*MGMT*) promoter is currently the strongest predictive biomarker of outcome and benefit from temozolomide-based treatment of GBM [8]. In 2008, Gorlia *et al.* integrated biological and clinical prognostic factors and their independent and combined predictive powers into nomograms for GBM patients treated with the standard regimen [9]. These nomograms can be used to predict an individual patient's median survival and the probability of survival at two years. These nomograms can be of interest in patient counselling and in the design and interpretation of clinical trials. However, the authors stressed a lack of statistical power in their subgroup analysis of patients who had an available *MGMT* promoter methylation status (n = 103).

In this study, we retrospectively analysed 399 GBM patients treated with the standard regimen (intention-to-treat). We identified the main clinical prognostic factors in this cohort and compared our results with those of the EORTC (European Organisation for Research and Treatment of Cancer) and NCIC (National Cancer Institute of Canada) trial 26981-22981/CE.3. We propose an updated nomogram intended to predict the survival time of an individual GBM patient. In a previous study on 50 GBM patients treated with the standard regimen, we showed that the methylation status of the diacylglycerol kinase iota gene (*DGKI*) promoter identified patients with *MGMT*-methylated tumors that responded poorly to the standard regimen [10]. The role of *DGKI* and the functional consequences of its methylation status have never been investigated in gliomas but *DGKI* regulates Ras signalling, an oncogenic pathway frequently altered in GBM [11], [12]. We further evaluated the potential predictive value of *DGKI* methylation status in the context of both *MGMT*-methylated and intention-to-treat populations.

## Materials and Methods

### Patients and tissue samples

This multi-center retrospective cohort included 399 patients treated in the Departments of Neurosurgery/Neuro-oncology of Angers (n = 28), Marseille (n = 52), Paris-Salpêtrière (n = 227), Rennes (n = 50), and Poitiers (n = 42) between 2006 and 2011. The inclusion criteria were as follows: 1) patients aged 18 years or more, 2) diagnosis of a primary GBM (WHO grade IV), 3) detailed clinical information at diagnosis and during follow-up, 4) treatment with radiotherapy and concurrent/adjuvant temozolomide (standard regimen), and 5) availability of tumor tissue with informed consent in accordance with French regulations and the Helsinki Declaration. All patients included in this study fulfilled the inclusion criteria. Particularly, all patients received a radio-chemotherapy regimen in accordance with the standard of care. Follow-up for included patients ranged from 24 days to 5.2 years (median, 15.5 months). Tumor samples were snap-frozen immediately after resection and stored in tumor banks under the following authorization numbers: (Centre de Ressources Biologiques, DC-2011-1467, Angers), 2008/70 (AP-HM Tumor Bank, Marseille), DC 2009-957 (OncoNeuroTheque Salpêtrière, Paris), DHOS/2004/04056 (Hospital Tumor Bank, Poitiers), and AC-2010-77 (Centre de Ressources Biologiques, Rennes). The extent of surgery was evaluated with an enhanced magnetic resonance imaging (MRI) performed within 24 hours after the resection. All samples presented at least 70% of tumor cells. For each tumor sample, DNA was extracted using the NucleoSpin Tissue Kit (Macherey Nagel) according to the manufacturer's instructions. The quality of the DNA samples was assessed by electrophoresis on a 1% agarose gel. Only high quality genomic DNAs were selected for further analyses.

Two patient populations were considered in this study: the population of all included patients (population 1) and the subgroup of *MGMT*-methylated patients (population 2). Population 1 was used to identify the main clinical prognostic factors and to compare our results with those of the EORTC and NCIC trial 26981-22981/CE.3 [9]. Population 2 was studied to evaluate the strength and importance of these prognostic factors after conventional *MGMT* stratification. The effect of *DGKI* promoter methylation status on the prognosis of GBM patients assigned to standard treatment was also evaluated in *MGMT*-methylated patients. Patients from population 2 were randomly assigned to a training cohort and a validation cohort of equal sizes in a randomized block design stratified by the hospital center. Table 1 shows the demographic and clinical characteristics of the patients included in this study.

**Table 1:** Patients demographic and clinical characteristics.

Characteristics		Population 1 (n = 399)	EORTC cohort* (n = 103)	Population 2 training (n = 86)	Population 2 validation (n = 89)
Age (years)					
	Median	59		57	59
	Range	21–88		29–88	26–80
Age - no. (%)					
	≤50	95 (24)	44 (43)	23 (27)	22 (25)
	51–60	130 (33)	40 (39)	30 (35)	26 (29)
	>60	174 (44)	19 (18)	33 (38)	41 (46)
Sex - no. (%)					
	Women	161 (40)	38 (37)	41 (48)	41 (46)
	Men	238 (60)	65 (63)	45 (52)	48 (54)
KPS (%)					
	Median	80		80	80
	Range	40–100		40–100	40–100
KPS - no. (%)					
	≤70	37 (9)		10 (12)	10 (11)
	>70	331 (83)		69 (80)	72 (81)
	Missing	31 (8)		7 (8)	7 (8)
Extent of surgery - no. (%)					
	Biopsy	30 (8)	0 (0)	10 (12)	5 (6)
	Partial resection	140 (35)	56 (54)	25 (29)	34 (38)
	Complete resection	220 (55)	47 (46)	50 (58)	49 (55)
	Missing	9 (2)	0 (0)	1 (1)	1 (1)
IDH1 mutational status - no. (%)					
	Mutated	18 (5)		5 (6)	8 (9)
	Wild-type	364 (91)		80 (93)	77 (87)
	Missing	17 (4)		1 (1)	4 (4)
MGMT methylation status - no. (%)					
	Methylated	175 (44)	45 (44)	86 (100)	89 (100)
	Unmethylated	224 (56)	58 (56)	0 (0)	0 (0)
DGKI methylation status - no. (%)					
	Methylated	95 (24)		22 (26)	21 (24)
	Ummethylated	304 (76)		61 (74)	68 (76)
Overall survival - mo					
	Median	19.1		29.6	30.2
	95% CI	17.1–20.8		22.5–46.7	24.1–46.8
Progression-free survival - mo					
	Median	10.8		15.2	15.6
	95% CI	10.1–11.9		13.8–19.1	13.1–23.4

*EORTC and NCIC trial 26981-22981/CE.3 population 3: GBM patients who underwent partial or complete resection and were assigned temozolomide and radiotherapy in the presence of an *MGMT* promoter methylation assessment.

### *IDH1* mutation

Tumor DNA was screened for somatic mutations in *IDH1 codon 132* via exon 4 PCR amplification and direct sequencing as previously described [13]. Because *IDH1* mutation is sufficient to establish the “glioma-CpG island methylator phenotype” (G-CIMP), we did not take this phenotype into account in our analysis [14].

### DNA methylation analysis

DNA was bisulfite-modified using the EZ DNA Methylation Kit (Zymo Research). The methylation percentage (%met) of the *MGMT* promoter was measured using the PyroMark Q96 CpG MGMT kit (Qiagen) (average percentage of the five tested CpG sites). The *DGKI* promoter's %met was measured using VeraCode GoldenGate Methylation technology (Illumina Inc.) or by pyrosequencing. PCR and pyrosequencing primers were designed using the Pyrosequencing Assay Design Software (Qiagen). The primers and PCR conditions are given in Figure S1. The reproducibility of the pyrosequencing assays was assessed on a subset of 21 patients for *MGMT* (Pearson correlation coefficient r = 0.999, p<1e-08) and of 26 patients for *DGKI* (Pearson correlation coefficient r = 0.994, p<1e-08). The consistency between VeraCode GoldenGate Methylation technology and pyrosequencing was assessed on 24 patients for DGKI (Pearson correlation coefficient r = 0.89, p<1e-08). The assessment of MGMT and DGKI %met was conducted at the Rennes hospital. For MGMT, we used the 8% methylation threshold defined on an independent data set by Quillien *et al.* [15]. The DGKI methylation threshold of 28% was determined in the training cohort using the *risksetROC* R package (AUC = 0.61).

### Statistical analyses

Overall survival (OS) and progression-free survival (PFS) were estimated using the Kaplan-Meier method. Comparisons between survival groups were performed using a log-rank test for binary variables, and a log-rank trend test for ordered categories. Cox proportional hazard models provided estimates of the hazard ratios (HRs). From these tests, variables with *p*-values less than 5% were candidates for the multivariate analyses. In population 1, the Cox proportional hazards model was then used with forward stepwise model selection. We have checked that no evidence of violation of the proportional hazards assumption was found. The probability of inclusion of a factor in the multivariate model was estimated by using the bootstrap resampling technique as described in Gorlia *et al*. [9]. All tests were adjusted for hospital center. Analyses were carried out using the *survival* R package.

A nomogram was developed for population 1 to predict each patient's median survival and probability of survival at two years, taking into account their clinical characteristics. Variables with a probability of inclusion higher than 90% based on 1000 bootstrap samples were included in the final model. The definition of two risk groups (high/low) was based on the value of the linear predictor underlying the nomogram; values greater than or equal to zero were assigned to the high-risk group, and negative values were assigned to the low-risk group (the total points cut-off between high and low-risk is the value matching a linear predictor value equal to zero). The accuracy of predictions was assessed by estimating the model's calibration and discrimination measured by the Concordance index corrected for optimism (C-index). The nomogram was built using the *rms* R package.

## Results

### Survival analysis of all included patients (population 1)

In population 1, univariate Cox analyses showed that age, Karnofsky performance status (KPS), extent of surgery, *IDH1* mutational status, *MGMT* promoter methylation status, and *DGKI* promoter methylation status were significantly associated with OS (Table 2). These variables remained significantly associated with OS in the multivariate Cox analyses (Table 3). The final multivariate Cox model used to build the nomogram included age, KPS, extent of surgery, *IDH1* mutational status, and *MGMT* promoter methylation status. This model was associated with a C-index corrected for optimism of 68%.

**Table 2:** Univariate analyses of survival prognostic factors.

		Population 1 (n = 399)			Population 2 (n = 175)					
					Training cohort (n = 86)			Validation cohort (n = 89)		
		Median survival, months (95% CI)	HR (95% CI)	p	Median survival, months (95% CI)	HR (95% CI)	p	Median survival, months (95% CI)	HR (95% CI)	p
Age (years)										
	≤50	33.1 (24.2–56.7)	..	<0.001	42.5 (33.1-N)	..	0.01	NA (37.8-N)	..	0.01
	51-60	19.3 (17.1–24.3)	1.4 (1.2–1.7)	..	31.4 (19.7-N)	1.7 (1.1–2.6)	..	30.2 (26.2-N)	1.8 (1.1–2.8)	..
	>60	15.6 (14.0–18.0)	..	..	20.1 (16.3-N)	..	..	16.5 (14.4–37.2)	..	..
Sex										
	Women	18.0 (16.3–26.0)	1.0	NS	34.8 (25.0-N)	1.0	NS	34.4 (17.8–N)	1.0	NS
	Men	19.3 (17.1–21.1)	1.2 (0.9–1.6)	..	24.6 (19.7-N)	1.6 (0.8–3.3)	..	27.5 (21.5–46.8)	0.9 (0.5–1.7)	..
KPS (%)										
	≤70	11.7 (10.2–16.9)	1.0	<0.001	19.3 (9.5-N)	1.0	0.002	13.3 (10.9-N)	1.0	0.002
	>70	19.8 (18.3–24.1)	0.5 (0.3–0.7)	..	33.1 (26.2–52.6)	0.1 (0.04–0.5)	..	34.4 (26.2-N)	0.2 (0.1–0.6)	..
Extent of surgery										
	Biopsy	14.5 (11.3–34.8)	..	0.002	14.5 (11.4-N)	..	0.01	20.3 (20.3-N)	..	NS
	Partial	19.2 (17.1–24.1)	0.7 (0.6–0.9)	..	19.9 (19.1-N)	0.6 (0.4–0.9)	..	37.8.1 (21.5-N)	0.8 (0.5–1.5)	..
	Complete	19.6 (17.1–22.3)	..	..	33.1 (25.0-N)	..	..	27.5 (22.3-N)	..	..
IDH1 mutational status										
	Mutated	56.7 (38.6-N)	1.0	<0.001	56.7 (N-N)	1.0	NS	NA (38.6-N)	1.0	NS
	Wild-type	18.3 (16.6–19.8)	7.8 (2.5–24.3)	..	27.8 (21.1–42.5)	4.4 (0.6–33.8)	..	24.8 (20.3–37.8)	5.3 (0.7–39.5)	..
MGMT methylation status										
	Methylated	30.2 (24.8–37.8)	1.0	<0.001						
	Unmethylated	14.9 (13.8–16.9)	3.1 (2.3–4.2)	..						
DGKI methylation status										
	Methylated	16.9 (14–19.9)	1.0	0.004	19.9 (9.9–N)	1.0	<0.001	16.5 (13.3-N)	1.0	0.01
	Unmethylated	19.6 (17.8–24.1)	0.7 (0.5–0.9)	..	34.8 (27.8–52.6)	0.3 (0.1–0.5)	..	37.8 (26.2-N)	0.4 (0.2–0.8)	..

NA = not available; N = not enough events to calculate upper 95% CI boundary; NS = not significant. Age, KPS and extent of surgery are treated as ordinal variables.

**Table 3:** Multivariate analyses of survival prognostic factors.

		Population 1 (n = 399)	
		HR (95% CI)	p (%inclusion)
Age (years)			
	≤50	..	0.006 (92)
	51–60	1.3 (1.1–1.6)	..
	>60	..	..
Karnofsky performance status (%)			
	≤70	1.0	<0.001 (99)
	>70	0.4 (0.2–0.6)	..
Extent of surgery			
	Biopsy	..	<0.001 (98)
	Partial resection	0.6 (0.5–0.8)	..
	Complete resection	..	..
IDH1 mutational status			
	Mutated	1.0	0.02 (94)
	Wild-type	4.1 (1.3–13.3)	..
MGMT methylation status			
	Methylated	1.0	<0.001 (100)
	Unmethylated	3.0 (2.2–4.2)	..
DGKI methylation status			
	Methylated	1.0	0.03 (74)
	Unmethylated	0.7 (0.5–1.0)	..

NA = not available; N = not enough events to calculate upper 95% CI boundary; NS = not significant. For ordered categorical factors, the first value is the reference.

**Figure 1:**
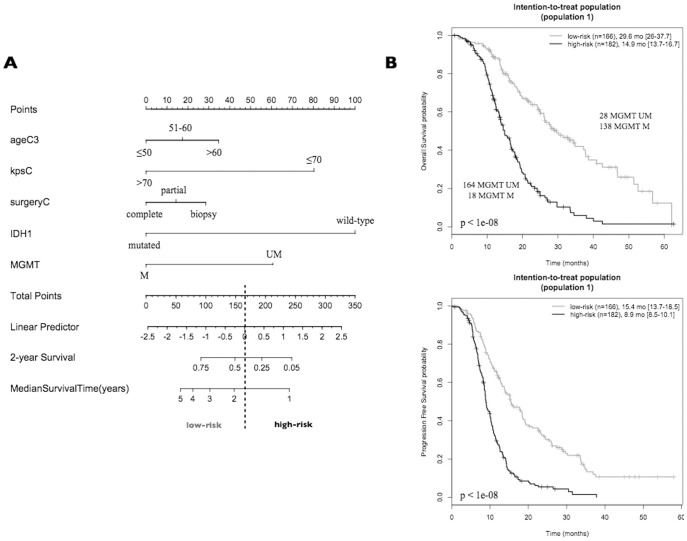
GBM patients assigned to standard treatment (population 1). (A) Nomogram for predicting median survival, probability of survival at two years, and risk category. The total number of points for each patient is obtained by summing the points for each of the individual factors in the nomogram. The median survival and probability of survival at two years for a given patient are obtained by drawing a vertical line from the “total points” axis down to the outcome axes. The definition of two risk groups (high/low) is based on the value of the linear predictor underlying the nomogram: values greater than or equal to zero were assigned to the high-risk group, and negative values were assigned to the low-risk group. The total point value matching the null value of the linear predictor is 165. Example: a 55-year-old patient with a KPS of 80 and a partly resected/*IDH1* wild-type/*MGMT*-methylated tumor has a total prognostic score of 129 and is predicted to have a median survival of approximately 30 months and a 60% probability of surviving two years. This patient is assigned to the low-risk group. (B) Kaplan-Meier estimation of OS and PFS. M: methylated patients, UM: unmethylated patients, LR: low-risk patients, HR: high-risk patients, mo: month. The difference in survival between groups is reported (log-rank test *p*-value). The size and the median survival of each group are also specified.

Figure 1A shows the nomogram for population 1. The total number of points for each patient is obtained by summing the points for each of the individual factors in the nomogram. The median survival and probability of survival at two years for a given patient are obtained by drawing a vertical line from the “total points” axis down to the outcome axes. For example, a 55-year-old patient with a KPS of 80 and a partly resected/*IDH1* wild-type/*MGMT*-methylated tumor has a total prognostic score of 129 and is predicted to have a median survival of approximately 30 months and a 60% probability of surviving two years. Because the cut-off between high and low risk is 165 points, this patient is assigned to the low-risk group. Patients in the low-risk group had a median OS of 29.6 months (95% CI, 26.0–37.7), which was significantly longer than 14.9 months (95% CI, 13.7–16.7) for patients in the high-risk group (p<1e-08). A similar stratification was observed for the PFS (Figure 1B).

### Survival analysis of *MGMT*-methylated patients (population 2)

**Figure 2:**
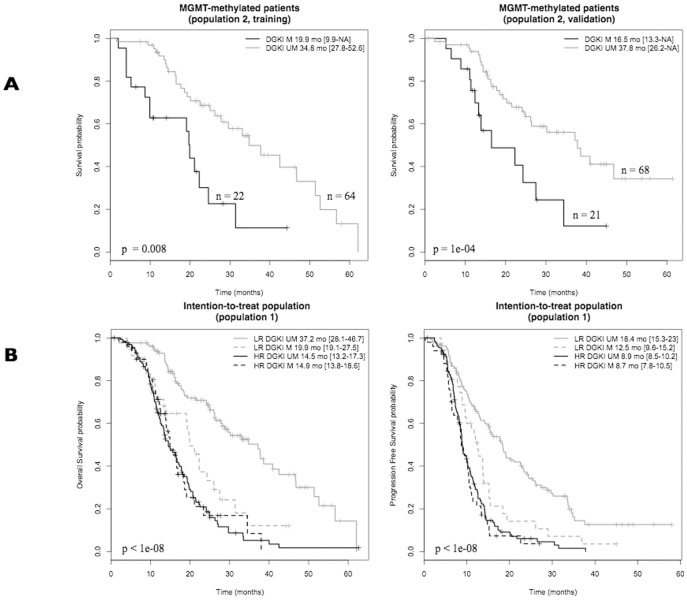
Prognostic value of *DGKI* methylation status. (A) *MGMT*-methylated GBM patients assigned to standard treatment (population 2). Kaplan-Meier estimation of OS in training and validation cohorts. (B) GBM patients assigned to standard treatment (population 1). Kaplan-Meier estimation of OS and PFS. M: methylated patients, UM: unmethylated patients, LR: low-risk patients, HR: high-risk patients, mo: month. The difference in survival between groups is reported (log-rank test *p*-value). The size and the median survival of each group are also specified.

In population 2, univariate Cox analyses showed that age, KPS, and *DGKI* promoter methylation status were significantly associated with OS in both the training and validation cohorts (Table 2). In the multivariate Cox analyses, *DGKI* promoter methylation status was the only variable with a probability of inclusion higher than 90% in the training cohort. *DGKI* methylation status stratified the *MGMT*-methylated patients into two groups with significant differences in OS (19.9 months *vs*. 34.8 months, p = 0.008, 16.5 months *vs*. 37.8 months, p = 1e-4, training and validation, respectively, figure 2A). The *MGMT* %met was not significantly different (Student's t-test) between the *DGKI*-methylated and *DGKI*-unmethylated patients (p = 0.23).

The OS and PFS of the *MGMT*-methylated and *DGKI*-methylated patients were not significantly different from the OS and PFS of the *MGMT*-unmethylated patients (19.7 months *vs.* 14.9 months, 12.0 months *vs.* 9.0 months, OS and PFS, respectively, Figure S2).

### Prognostic value of *DGKI* methylation status in risk groups (population 1)

The *DGKI* methylation status stratified the low-risk patients into two groups with significant differences in OS and PFS (37.2 months *vs*. 19.9 months, 18.4 months *vs*. 12.5 months, OS and PFS, respectively, figure 2B). The OS of low-risk and *DGKI*-methylated patients was not significantly different from the OS of high-risk patients (19.9 months *vs.* 14.9 months, p = 0.21). The *DGKI* methylation status did not stratify the subgroup of high-risk patients in the intention-to-treat population.

## Discussion

We studied a retrospective cohort of 399 GBM patients homogeneously treated with the standard regimen. The higher OS at 6 and 12 months observed in our cohort in comparison to the reference cohort of the EORTC trial [16] (Table S1) can be explained by an improvement of surgical practices (55% of complete resection in our cohort vs. 39% in EORTC trial), continuation of standard treatment despite pseudoprogression, and an earlier and/or easier access to bevacizumab treatment at recurrence for patients progressing after 2007. This cohort was used to identify the main clinical prognostic factors and to design a nomogram intended to predict the survival time of an individual patient. A nomogram-based stratification of the cohort identified two risk groups (high/low) with significantly different median survival. In the low-risk group, the *DGKI* promoter methylation status identified poorly responding patients.

The prognostic factors identified in our study were age, KPS, extent of surgery, *IDH1* mutational status, and *MGMT* promoter methylation status. This result is in agreement with the most consistently reported prognostic factors for GBM [2], [3], [4], [5], [6], [8]. From an individual patient's perspective, a nomogram offers a more tailored approach, taking into account their clinical characteristics. This can be of interest in patient counselling and in the design and interpretation of clinical trials. Therefore, we propose a nomogram based on the prognostic factors identified in our study. The nomogram can be used to predict an individual patient's median survival and probability of survival at two years. The DGKI methylation status was not included in this nomogram as it did not identify clinically relevant groups of patients in the intention-to-treat population. However, the interaction between MGMT and DGKI in a multivariate Cox model including all significant prognostic factors (age+KPS+surgery+IDH1+MGMT+DGKI+MGMT:DGKI) was significant (p = 0.0007). This indicated that the prognostic value of DGKI was to find in the context of the MGMT methylation status. A nomogram including this interaction can be found in Figure S3.

In 2008, Gorlia *et al.* proposed a nomogram for GBM patients who underwent either a partial or complete resection and were assigned to temozolomide and radiotherapy in the presence of a *MGMT* promoter methylation assessment (population 3 of the EORTC and NCIC trial 26981-22981/CE.3). Their nomogram includes *MGMT* promoter methylation status, Folstein Mini-Mental State Examination (MMSE) score, and WHO performance status [9]. We propose an updated version of this nomogram that includes not only *MGMT* promoter methylation status and Karnofsky performance score but also age, *IDH1* mutational status, and extent of surgery. In the EORTC trial, elderly patients (>70 years) were excluded, and the prognostic value of *IDH1* was not evident when the trial was designed. Furthermore, the authors discussed the restricted reliability of the extent of surgery in their study. Age and extent of surgery were nonetheless identified by Gorlia *et al.* in the population of GBM patients who underwent partial or complete resection and were assigned to temozolomide and radiotherapy without the knowledge of the *MGMT* promoter methylation status (population 2 of the EORTC trial). Because it was not routine to collect MMSE score in the neurosurgical units involved, we were unable to evaluate the prognostic value of these nomograms on our cohort. However, the nomogram proposed in the present study showed better performance evaluation (AUC) than MGMT status alone (0.71[0.65–0.78] vs. 0.65[0.59–0.71], p<1e-08). The potential skewing effect of treatment at recurrence on patient survival was not controlled; however, the validity of our PFS results indicated that our findings could be independent of these treatments. A recent study showed that the nomogram designed by Gorlia *et al.* for the intention-to-treat population (radiotherapy only or temozolomide and radiotherapy) was a poor predictor of an individual patient's survival because the standard of care has evolved since the EORTC trial [17]. Unfortunately, the nomograms proposed by Gorlia *et al.* for GBM patients who received the standard treatment (population 2 and population 3 of the EORTC trial) were not evaluated in the study by Parks *et al*. However, our study confirmed the appropriateness of Gorlia *et al.* findings as we also identified *MGMT* methylation status, performance status, age and extent of surgery in the nomogram for the population of GBM patients assigned to standard treatment.

A nomogram-based stratification of our cohort of primary GBM patients treated with the standard regimen identified two risk groups (high/low) with significantly different median survival. The low-risk group was almost exclusively composed of *MGMT*-methylated patients. Interestingly, our previous study showed that the methylation status of the *DGKI* promoter identified GBM patients with *MGMT*-methylated tumors who responded poorly to the standard regimen [10]. In this study, we have validated the prognostic value of *DGKI* methylation status for *MGMT*-methylated patients (population 2). However, this finding could be restricted by the limited size of the training and validation cohorts. We further evaluated the potential predictive value of *DGKI* methylation status on the intention-to-treat population (population 1). The methylation status of the *DGKI* promoter identified 22% of poorly responding patients in the low-risk group but had no prognostic value for high-risk patients. The role of *DGKI* and the functional consequences of its methylation status have never been investigated in gliomas but *DGKI* regulates Ras signalling, an oncogenic pathway frequently altered in GBM [11], [12]. Recently, Revill et al. showed that *DGKI* was hypermethylated in primary hepatocellular carcinoma and was re-expressed in liver cancer cell lines after exposure to reagents reversing DNA methylation [18]. This study suggests that *DGKI* expression is regulated by its promoter methylation. In GBM, we observed an anti-correlation between *DGKI* expression and methylation levels, in a private cohort and in the TCGA cohort (data not shown). Further functional studies on *DGKI* are clearly required.

Our results improve the conventional *MGMT* stratification of GBM patients receiving standard treatment. In particular, the *DGKI* methylation status identified poorly responding patients in the group of low-risk or *MGMT*-methylated patients. A retrospective study precluding the establishment of firm conclusions, these results need to be validated in a prospectively recruited cohort. They could however be of help in the interpretation of published or ongoing clinical trial outcomes and refine patient recruitment in the future.

## Supporting Information

Figure S1 **Primers and cycling conditions for the pyrosequencing analysis of DGKI_7.**
(TIFF)

Figure S2 **Prognostic value of** ***DGKI*** **methylation status for GBM patients assigned to standard treatment (population 1).** Kaplan-Meier estimation of OS and PFS. M: methylated patients, UM: unmethylated patients, mo: month. The difference in survival between groups is reported (log-rank test *p*-value). The size and the median survival of each group are also specified.
(TIFF)

Figure S3 **Nomogram including the interaction between MGMT and DGKI (population 1).**
(TIFF)

Table S1 **OS ans PFS - % (95% IC).**
(DOC)

## References

[1] Wen PY, Kesari S (2008) Malignant gliomas in adults. N Engl J Med 359: 492–507. 18669428 10.1056/NEJMra0708126

[2] Siker ML, Wang M, Porter K, Nelson DF, Curran WJ, et al (2011) Age as an independent prognostic factor in patients with glioblastoma: a Radiation Therapy Oncology Group and American College of Surgeons National Cancer Data Base comparison. J Neurooncol 104: 351–356. 21221714 10.1007/s11060-010-0500-6

[3] Stummer W, van den Bent MJ, Westphal M (2011) Cytoreductive surgery of glioblastoma as the key to successful adjuvant therapies: new arguments in an old discussion. Acta Neurochir (Wien) 153: 1211–1218. 21479583 10.1007/s00701-011-1001-x

[4] Filippini G, Falcone C, Boiardi A, Broggi G, Bruzzone MG, et al (2008) Prognostic factors for survival in 676 consecutive patients with newly diagnosed primary glioblastoma. Neuro Oncol 10: 79–87. 17993634 10.1215/15228517-2007-038 PMC2600841

[5] Ichimura K, Pearson DM, Kocialkowski S, Backlund LM, Chan R, et al (2009) IDH1 mutations are present in the majority of common adult gliomas but rare in primary glioblastomas. Neuro Oncol 11: 341–347. 19435942 10.1215/15228517-2009-025 PMC2743214

[6] Yan H, Parsons DW, Jin G, McLendon R, Rasheed BA, et al (2009) IDH1 and IDH2 mutations in gliomas. N Engl J Med 360: 765–773. 19228619 10.1056/NEJMoa0808710 PMC2820383

[7] Sanson M, Marie Y, Paris S, Idbaih A, Laffaire J, et al (2009) Isocitrate dehydrogenase 1 codon 132 mutation is an important prognostic biomarker in gliomas. J Clin Oncol 27: 4150–4154. 19636000 10.1200/JCO.2009.21.9832

[8] Stupp R, Hegi ME, Mason WP, van den Bent MJ, Taphoorn MJ, et al (2009) Effects of radiotherapy with concomitant and adjuvant temozolomide versus radiotherapy alone on survival in glioblastoma in a randomised phase III study: 5-year analysis of the EORTC-NCIC trial. Lancet Oncol 10: 459–466. 19269895 10.1016/S1470-2045(09)70025-7

[9] Gorlia T, van den Bent MJ, Hegi ME, Mirimanoff RO, Weller M, et al (2008) Nomograms for predicting survival of patients with newly diagnosed glioblastoma: prognostic factor analysis of EORTC and NCIC trial 26981-22981/CE.3. Lancet Oncol 9: 29–38. 18082451 10.1016/S1470-2045(07)70384-4

[10] Etcheverry A, Aubry M, de Tayrac M, Vauleon E, Boniface R, et al (2010) DNA methylation in glioblastoma: impact on gene expression and clinical outcome. BMC Genomics 11: 701. 21156036 10.1186/1471-2164-11-701 PMC3018478

[11] Regier DS, Higbee J, Lund KM, Sakane F, Prescott SM, et al (2005) Diacylglycerol kinase iota regulates Ras guanyl-releasing protein 3 and inhibits Rap1 signaling. Proc Natl Acad Sci U S A 102: 7595–7600. 15894621 10.1073/pnas.0500663102 PMC1140424

[12] The Cancer Genome Atlas Network (2008) Comprehensive genomic characterization defines human glioblastoma genes and core pathways. Nature 455: 1061–1068. 18772890 10.1038/nature07385 PMC2671642

[13] Kang MR, Kim MS, Oh JE, Kim YR, Song SY, et al (2009) Mutational analysis of IDH1 codon 132 in glioblastomas and other common cancers. Int J Cancer 125: 353–355. 19378339 10.1002/ijc.24379

[14] Turcan S, Rohle D, Goenka A, Walsh LA, Fang F, et al (2012) IDH1 mutation is sufficient to establish the glioma hypermethylator phenotype. Nature 483: 479–483. 22343889 10.1038/nature10866 PMC3351699

[15] Quillien V, Lavenu A, Karayan-Tapon L, Carpentier C, Labussiere M, et al.. (2012) Comparative assessment of 5 methods (methylation-specific polymerase chain reaction, methylight, pyrosequencing, methylation-sensitive high-resolution melting, and immunohistochemistry) to analyze O6-methylguanine-DNA-methyltranferase in a series of 100 glioblastoma patients. Cancer. 10.1002/cncr.27392 22294349

[16] Stupp R, Mason WP, van den Bent MJ, Weller M, Fisher B, et al (2005) Radiotherapy plus concomitant and adjuvant temozolomide for glioblastoma. N Engl J Med 352: 987–996. 15758009 10.1056/NEJMoa043330

[17] Parks C, Heald J, Hall G, Kamaly-Asl I (2013) Can the prognosis of individual patients with glioblastoma be predicted using an online calculator? Neuro Oncol. 10.1093/neuonc/not033 PMC3714148 23543729

[18] Revill K, Wang T, Lachenmayer A, Kojima K, Harrington A, et al. (2013) Genome-wide methylation analysis and epigenetic unmasking identify tumor suppressor genes in hepatocellular carcinoma. Gastroenterology 145: : 1424–1435 e1421–1425. 10.1053/j.gastro.2013.08.055 PMC3892430 24012984

