# Supplementary figures and images for: DGKI Methylation Status Modulates the Prognostic Value of MGMT in Glioblastoma Patients Treated with Combined Radio-Chemotherapy with Temozolomide
Source: PLoS One. 2014 Sep 18;9(9):e104455. doi: 10.1371/journal.pone.0104455 (PMC4169423; doi:10.1371/journal.pone.0104455)

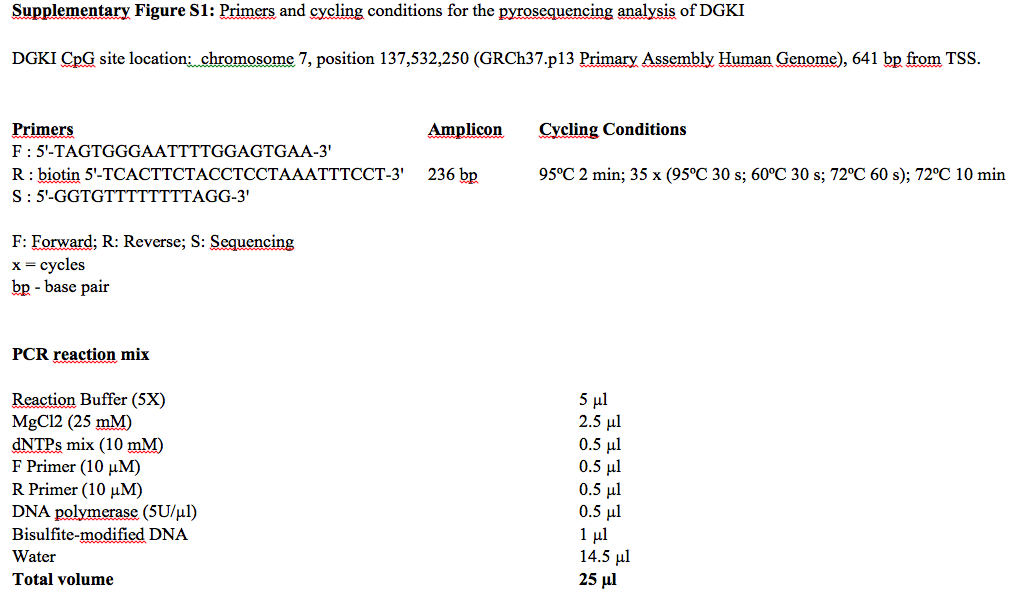

Supplement: Figure S1 — Primers and cycling conditions for the pyrosequencing analysis of DGKI_7. (TIFF) [file pone.0104455.s001.tiff]

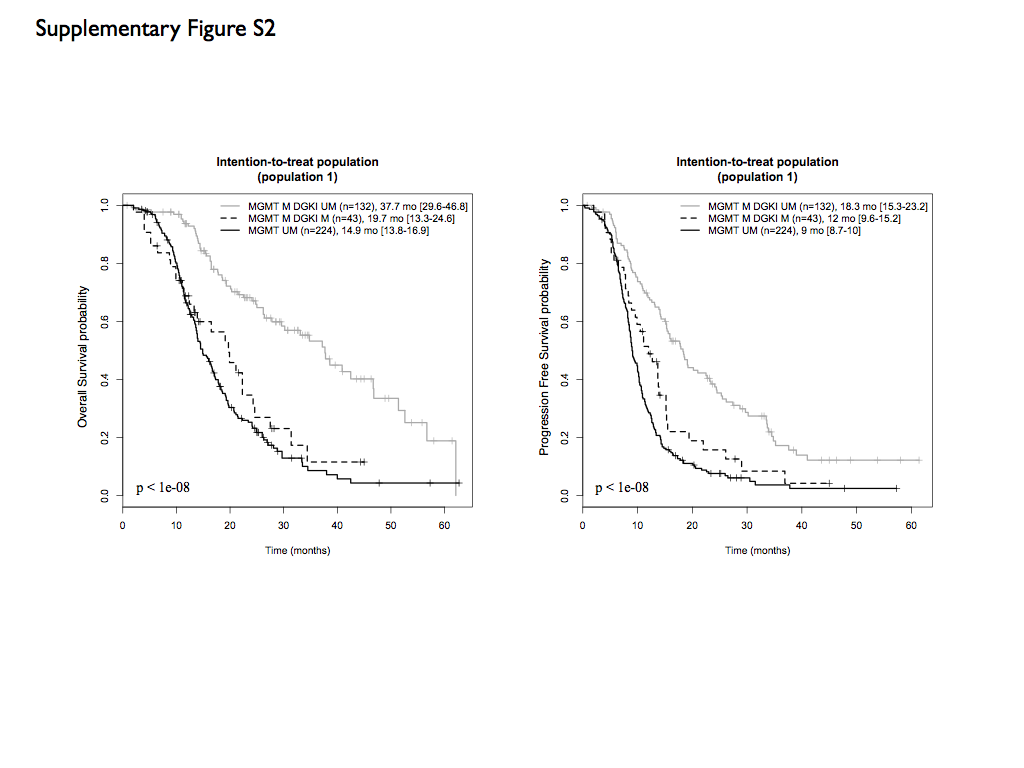

Supplement: Figure S2 — Prognostic value of DGKI methylation status for GBM patients assigned to standard treatment (population 1). Kaplan-Meier estimation of OS and PFS. M: methylated patients, UM: unmethylated patients, mo: month. The difference in survival between groups is reported (log-rank test p-value). The size and the median survival of each group are also specified. (TIFF) [file pone.0104455.s002.tiff]

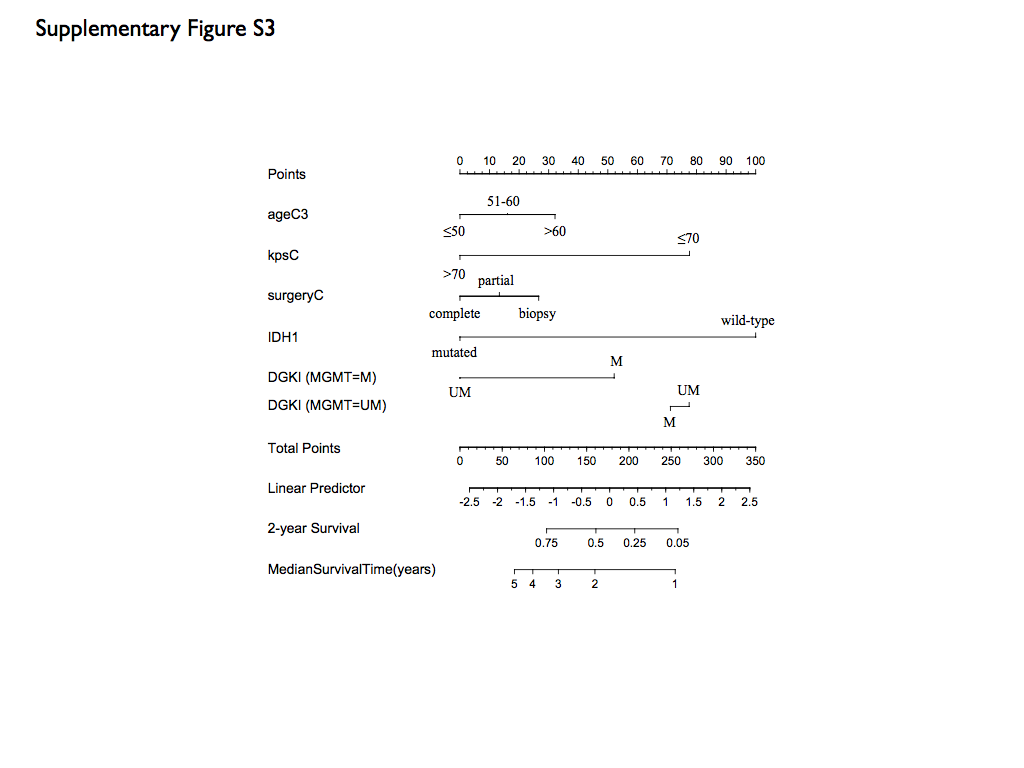

Supplement: Figure S3 — Nomogram including the interaction between MGMT and DGKI (population 1). (TIFF) [file pone.0104455.s003.tiff]
