# Supplementary material for: DGKI Methylation Status Modulates the Prognostic Value of MGMT in Glioblastoma Patients Treated with Combined Radio-Chemotherapy with Temozolomide
Source: PLoS One. 2014 Sep 18;9(9):e104455. doi: 10.1371/journal.pone.0104455 (PMC4169423; doi:10.1371/journal.pone.0104455)
Supplement: Table S1 — OS ans PFS - % (95% IC). (DOC) [file pone.0104455.s004.doc]

| **Supplementary Table S1:** OS ans PFS - % (95% IC) | | |
| --- | --- | --- |
|  |  |  |
|  |  |  |
| **OS** | **Population 1** | **EORTC trial** |
| At 6 months | 93.7 [91.3-96.1] | 86.3 [82.3-90.3] |
| At 12 months | 67.9 [63.3-72.5] | 61.1 [55.4-66.7] |
| At 18 months | 39.8 [35.0-44.6] | 39.4 [33.8-45.1] |
| At 24 months | 26.8 [22.5-31.1] | 26.5 [21.2-31.7] |
|  |  |  |
|  |  |  |
| **PFS** | **Population 1** | **EORTC trial** |
| At 6 months | 83.2 [79.5-86.9] | 53.9 [48.1-59.6] |
| At 12 months | 40.8 [36.0-45.7] | 26.9 [21.8-32.1] |
| At 18 months | 21.3 [17.3-25.3] | 18.4 [13.9-22.9] |
| At 24 months | 13.8 [10.4-17.2] | 10.7 [7.0-14.3] |
